# The Effects of Heat Treatment on the Impact Toughness and Fracture of Selective Laser-Melted Corrax Maraging Stainless Steel

**DOI:** 10.3390/ma18051150

**Published:** 2025-03-04

**Authors:** Ming-Hsiang Ku, Shu-Wei Ku, Chien-Lun Li, Shih-Hsien Chang, Ming-Wei Wu

**Affiliations:** 1 Department of Materials and Mineral Resources Engineering, National Taipei University of Technology, Taipei 10608, Taiwan; lenno6622@yahoo.com.tw (M.-H.K.); tienanman0604@gmail.com (S.-W.K.); changsh@ntut.edu.tw (S.-H.C.); 2 Voestalpine Technology Institute (Asia) Co., Ltd., Nantou 54041, Taiwan; allen.licl@voestalpine.com

**Keywords:** selective laser melting, heat treatment, building direction, maraging stainless steel, impact energy

## Abstract

In additive manufacturing (AM) metallic materials, heat treatment (HT) is a common process for modifying the unstable and anisotropic microstructure. Selective laser melting (SLM) Corrax maraging stainless steel is a novel material that has been applied in mold materials with conformal cooling channels in industry. However, the influences of HTs on the various mechanical properties of SLM Corrax steels are still not fully clarified. The aim of this research was thus to clarify the effects of solution treatment (S) and integrated solution-aging treatment (SA) on the hardness and impact toughness of SLM Corrax maraging stainless steel. Furthermore, to identify the roles of building direction (BD) on the hardness and impact toughness, parallelly built (P) and vertically built (V) SLM Corrax steels were fabricated and compared. The microstructures were examined by X-ray diffraction, electron backscatter diffraction, and electron probe micro-analysis, and to observe the fracture surface, scanning electron microscopy was used. The results showed that both the impact energies and apparent hardnesses were dominated by the HT. S treatment simultaneously decreased the impact energies and apparent hardnesses. SA treatment increased the apparent hardnesses but decreased the impact energies. BD did not apparently affect either the hardness or the toughness. Furthermore, the percentage of austenite did not affect the impact energies of the various material conditions. In the SA condition, the apparent hardnesses of P and V specimens were 49.9 HRC and 49.3 HRC, respectively. The impact energies of SA-P and SA-V specimens were 20 J and 17 J, respectively. The low anisotropy of SA specimens in hardness and toughness can be attributed to the weak texture and is advantageous to the material’s stability during service.

## 1. Introduction

Selective laser melting (SLM) is currently one of the most extensively applied additive manufacturing (AM) methods for various alloys [1,2,3,4,5,6,7]. Molds made by traditional mechanical processes (such as casting or forging) have a long manufacturing cycle and cannot be produced in complex shapes. In addition, the finished mold requires a long cooling time, which can easily lead to warpage [8,9]. Using the SLM process to manufacture molds reduces the processing time and enables the production of molds with more complex geometries. These advantages solve the problems of air traps and other issues in the mold production process [10,11]. The porosity of SLM alloys can be lower than 1% with suitable combinations of processing parameters [5,12,13].

Corrax^®^ and CX maraging stainless steels have similar compositions and were developed by Uddeholm AB and EOS GmbH, respectively. These low-carbon, precipitation-hardening types of maraging stainless steels are known for their high-performance, good anti-corrosion behavior, high hardness, and high mechanical strength [12,13,14,15,16,17]. These properties make them suitable for application in areas such as plastic injection molding (PIM), aviation, healthcare, and marine industries [15,18,19,20]. The major focus in research on Corrax (or CX) steel has been the influences of heat treatment (HT) on the microstructure, mechanical performances, and anti-corrosion behavior [14,15,16,20,21,22,23].

Zhang et al. [21] showed that excellent mechanical properties were achieved in SLM CX steel after integrated solution-aging treatment (SA) because many small β-NiAl particles were precipitated in the matrix. Afkhami et al. [13] found that, under tensile loading, SLM CX steel exhibits anisotropic properties. After HT, the anisotropy in the strength is significantly reduced. Wu et al. [14] reported that, in SLM Corrax steel, a weak texture can be identified in different material conditions. However, the building direction (BD) and HT do not apparently modify the anisotropy in tensile performances. After SA treatment, the yield strength (YS) and ultimate tensile strength (UTS) of SLM Corrax steel are sufficiently improved without an obvious sacrifice in elongation.

As reported in many studies [24,25,26,27,28], microstructures with obvious preferred orientations are generated during the SLM process. Recently, some studies [13,21,22,23,29] have found that, for various SLM alloys, HT can not only change the preferred orientation and anisotropic tensile properties but also eliminate residual stress, further improving the mechanical properties of the alloys and increasing their industrial application. In addition, the BD of the SLM process is a major factor that dominates the microstructure and mechanical performance. There are obvious differences in the microstructures and tensile performances of SLM CX stainless steel specimens fabricated by changing BDs [13,15]. However, Wu et al. [14] reported that the BD and HT do not apparently change the texture of SLM Corrax.

Another important performance of a structural material is impact toughness. The impact energy of parallelly built specimens of SLM Ti-6Al-4V alloy is higher than that of vertically built specimens [27,28]. This phenomenon is attributed to the many disc-shaped pores generated during the SLM process. Chang et al. [22] indicated that after SA heat treatment, the hardness of SLM CX steel significantly raises. However, SA heat treatment also reduces the impact energy of the steel from 83.8 J to just 5.3 J. According to the above background, the mechanical performances of SLM Corrax and CX are complex. However, the roles of HTs and BD in the various performances of SLM Corrax steels are still not fully identified, particularly the impact toughness. There are still knowledge gaps regarding the correlation between the microstructure, such as phase constituent and porosity, and the mechanical performances of the SLM Corrax or CX, and this uncertainty largely inhibits their application in industry. Therefore, the aim of this research was to understand the effects of heat treatments, including solution treatment (S) and SA, on the impact toughness and hardness of SLM Corrax maraging stainless steel. Furthermore, the SLM Corrax steels were parallelly built (P) and vertically built (V) to clarify the role of BD. The microstructures and fracture surfaces were analyzed to identify the effects of HT and BD on the toughness and hardness. The relationship between the microstructure and impact toughness and hardness was examined in this study.

## 2. Experimental Procedures

Commercial gas-atomized Corrax powder was applied in this study [14]. The chemical composition (wt%) provided by the raw material supplier was Fe-12.0Cr-9.2Ni-1.4Mo-1.6Al-0.3Mn-0.3Si-0.03C. SLM Corrax stainless-steel specimens were produced with an SLM machine (EOS M290, EOS GmbH, Krailling, Germany). The SLM parameters can be found in the literature [14]. To investigate the influences of BD, the impact specimens (55 mm × 10 mm × 10 mm) were parallelly built and vertically built. Impact specimens with a 45° V-notch and a notch depth of 2 mm were produced as per ASTM standard E23-12c [30]. The lengths of the P and V specimens were perpendicular and parallel, respectively, to the BD (Z axis).

**Figure 1:**
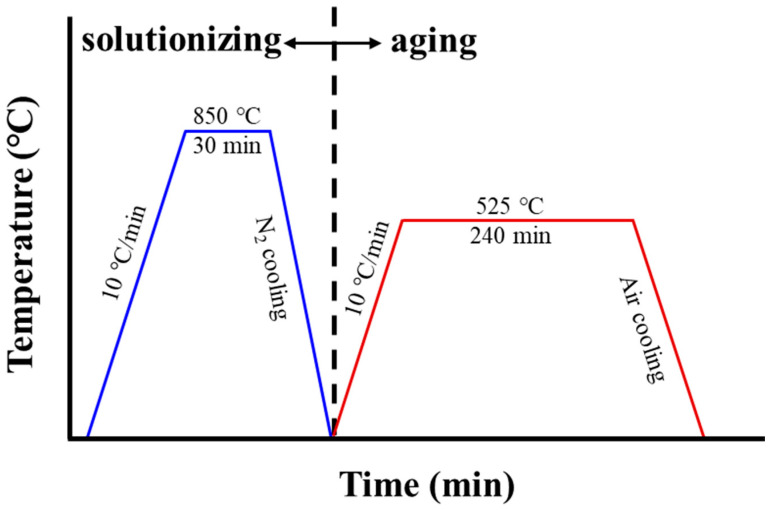
Schematic of HT processes in this study.

To improve the microstructures and mechanical performances of SLM Corrax specimens, two types of HTs were performed, including S and SA. Zhang et al. [21] reported that the hardness of SLM CX steels decreases by raising the solution temperature from 800 °C to 1000 °C. In addition, too high a temperature (900 °C) and too long a time (1.5 h) lead to microstructural coarsening and the decrease in hardness. Hence, solution treatment at 850 °C for 30 min can obtain better mechanical properties [21]. On the other hand, after SA treatment at 530 °C for 3 h, the hardness of SLM CX steel reaches its peak [21]. In this study, the parameters for the S and SA treatments were suggested and optimized by the manufacturer of the raw Corrax powder, Uddeholm AB, Hagfors, Sweden. A schematic illustrating the HT processes is provided in Figure 1, and the detailed parameters can be found in the literature [14]. In this study, as-built specimens were designated as A specimens. Specimens treated with the S and SA processes were designated as S and SA specimens, respectively. The parallelly built/vertically built A, S, and SA specimens were named A-P/A-V, S-P/S-V, and SA-P/SA-V, respectively.

To clarify the crystal structure and orientation, the A, S, and SA specimens were analyzed by an X-ray diffractometer (XRD, Rigaku MiniFlex600-C, Rigaku Corporation, Tokyo, Japan) and electron backscatter diffraction (EBSD, NordlysNano, Oxford Instrument, Oxford, UK). The software AZtecCrystal v2.2 was used for EBSD data processing. To clarify the element distribution, electron probe micro-analysis (EPMA, JXA-8200SX, JEOL, Tokyo, Japan) was applied to conduct mapping and quantitative analysis. The EBSD specimens were ground and polished without etching. The EPMA specimens were etched with an etchant (5 g copper chloride + 100 mL HCl + 100 mL alcohol). A Rockwell hardness tester (8150SK, Indentec Hardness Testing Machines Ltd., Worcester, UK) was used to measure the apparent hardness. Moreover, the SLM Corrax steels were examined with a micro-Vickers hardness tester (MMX-T, MATSUZAWA, Tokyo, Japan) with a loading of 0.98 N to determine the micro-hardness. The reported hardnesses are the averages of five measurements. Impact testing was performed with a Charpy impact machine (Shimadzu Corporation, Kyoto, Japan) to measure the impact energy as functions of material condition and BD. The reported data on the impact energies of each specimen condition are averages of three specimens. A JEOL JSM-6510LV scanning electron microscope was applied to observe the impact fracture surfaces and thereby determine the fracture modes.

## 3. Results and Discussion

### 3.1. Microstructure and Elemental Distribution

**Figure 2:**
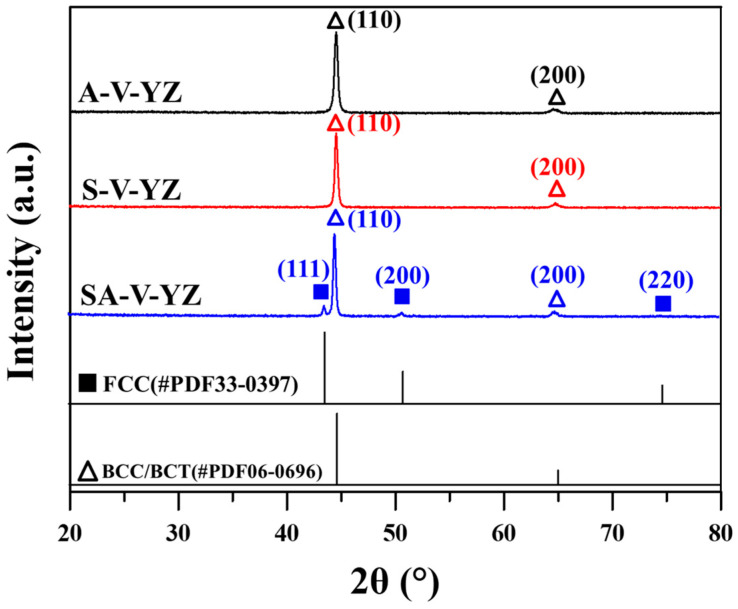
The XRD patterns on the YZ planes of V specimens in different material conditions.

**Figure 3:**
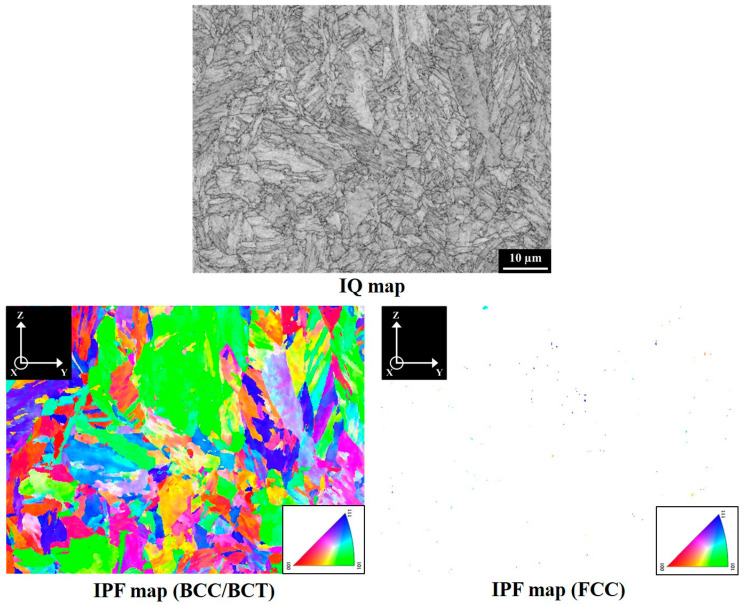
The EBSD microstructure of an A-V specimen.

**Figure 4:**
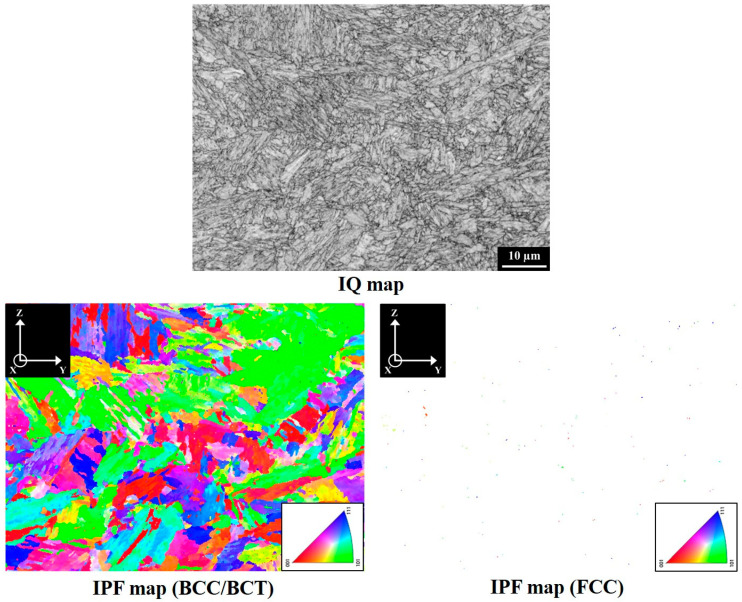
The EBSD microstructure of an S-V specimen.

**Figure 5:**
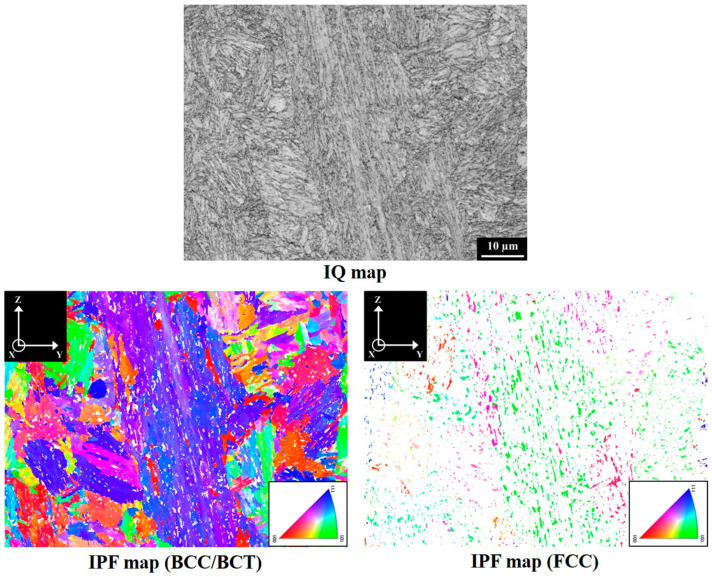
The EBSD microstructure of an SA-V specimen.

Figure 2 presents the XRD patterns on the YZ planes of V specimens in different material conditions. The results indicated that the SA-V specimens consisted of body-centered tetragonal (BCT)/body-centered cubic (BCC) and face-centered cubic (FCC) crystal structures. However, only the diffraction peaks corresponding to the BCC/BCT crystal structures were identified in both the A-V and S-V specimen. These findings indicate that SA treatment increased the fraction of austenite in the A-V specimen. The lattice constants of BCC/BCT structures in the A-V, S-V, and SA-V specimens were 2.873 Å, 2.867 Å, and 2.869 Å, respectively. Figure 3, Figure 4 and Figure 5 show the EBSD findings on the YZ planes of the A-V, S-V, and SA-V specimens, respectively. The image quality (IQ) map and inverse pole figure (IPF) map are also shown in Figure 3, Figure 4 and Figure 5. From the figures, the matrix of the A-V, S-V, and SA-V specimens was identified as a BCC or BCT phase, and a lath-like structure was found. Several studies have found that lath martensite is the major phase of SLM Corrax or CX steel [12,14,15,16,31]. Moreover, minor proportions of austenite were present in the A-V, S-V, and SA-V specimens. Table 1 presents the EBSD results as functions of specimen situation, BD, and plane analyzed [14]. In general, the S treatment reduced the amount of austenite because the austenite dissolved into the matrix during the S treatment. Moreover, the SA process apparently raised the fraction of austenite because of the formation of reverted austenite [14]. The EBSD findings corresponded well with the XRD findings. Furthermore, the microstructures in the A-V, S-V, and SA-V specimens did not exhibit an obvious preferred orientation, as shown in Figure 3, Figure 4 and Figure 5.

**Figure 6:**
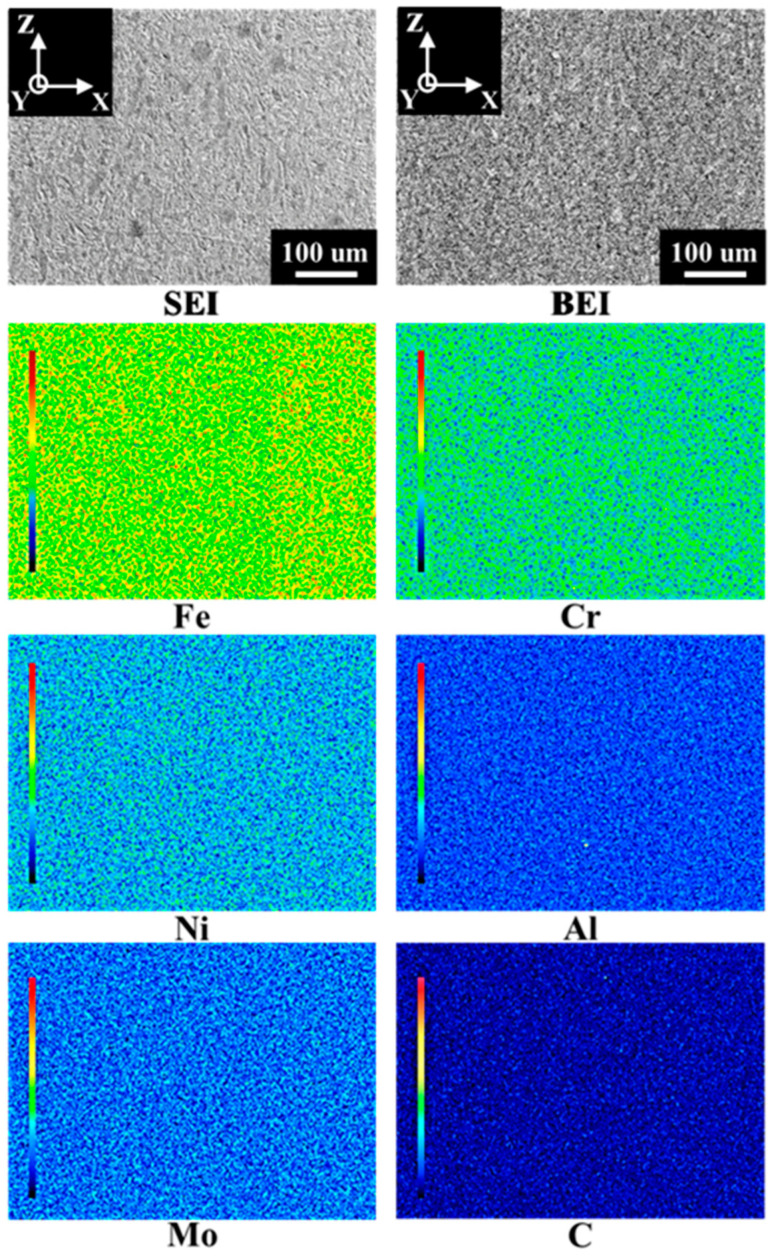
The EPMA elemental maps in an A-V specimen.

To examine the elemental distribution, EPMA was used to analyze the elemental maps and perform quantitative analyses. As shown in Figure 6, in the representative areas of an A-V specimen, each element was evenly distributed, with no apparent segregation. Furthermore, each metallic element was quantitatively analyzed in six randomly selected regions, as listed in Table 2. The deviations in chemical composition in each area were minor. The results in Table 2 corresponded well to those in Figure 6. It must be noted that the elemental segregation between the martensitic matrix and austenite or NiAl precipitates cannot be identified in Figure 4, Figure 5 and Figure 6 due to the spatial resolutions of EPMA and EBSD. Several studies [15,21,22,32] have used transmission electron microscopy (TEM) to identify the precipitates in SLM Corrax or CX. They found that nano-scale β-NiAl precipitates are generated and are dispersed inside the martensitic matrix after SA treatment.

### 3.2. Hardness and Impact Energy

**Figure 7:**
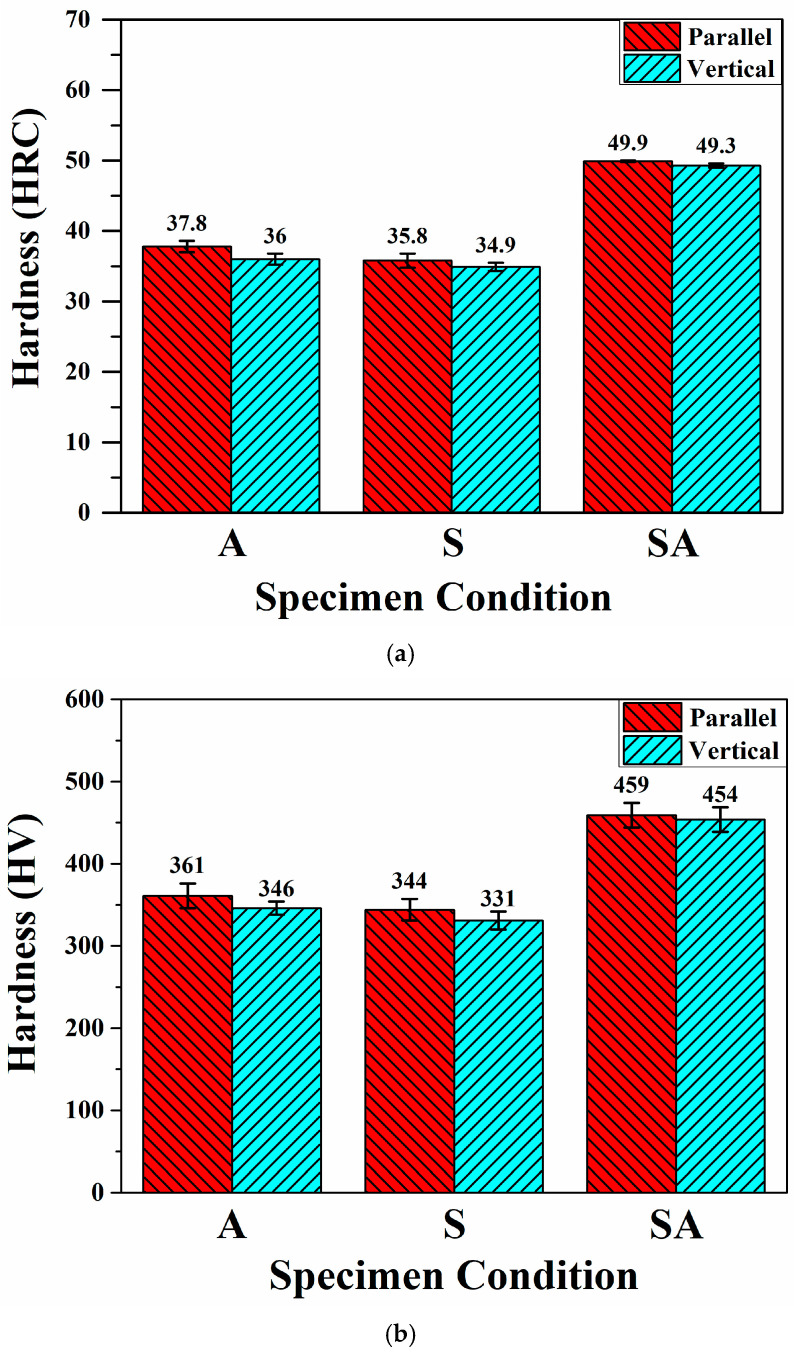
(**a**) The apparent hardnesses and (**b**) micro-hardnesses of the specimens in various conditions.

To understand the effects of BD and HT on hardness and impact toughness, hardness and impact testing were performed on various specimens. Figure 7 presents the hardnesses as functions of BD and specimen condition. The hardnesses of the as-built specimens were similar to those of conventional wrought Corrax steel [33]. The effects of the various HT conditions were different. After S treatment, the apparent hardnesses of the A specimens slightly decreased. This trend was likely due to the decrease in the percentage of austenite after S treatment. In contrast, SA treatment greatly improved the apparent hardnesses. The apparent hardnesses of SA-P and SA-V specimens were increased to 49.9 HRC and 49.3 HRC, respectively. These significant increases in apparent hardness after SA treatment can be ascribed to precipitation strengthening by β-NiAl particles with sizes of 5.6 nm to 50 nm that precipitated in the martensitic matrix [15,21,22,32]. Precipitation strengthening contributed to the obvious increase in hardness after the SA treatment despite the higher amount of austenite after the treatment. On the other hand, in the same material condition, there was no obvious difference in the apparent hardnesses of P and V specimens, indicating that the BD had no obvious role in the hardness. The effects of HT and BD on the micro-hardness corresponded well to those on the apparent hardness.

**Figure 8:**
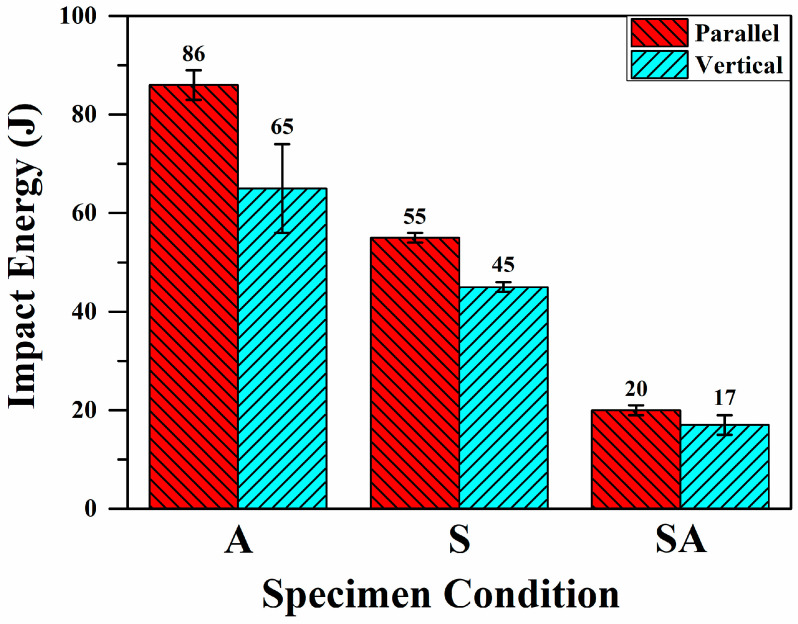
The impact energies of the specimens in various conditions.

Figure 8 shows the effects of BD and HT on the impact energies of A specimens. Among the impact energies for each condition, the A specimens exhibited the highest ones; those of A-P and A-V specimens were 86 J and 65 J, respectively. In addition, after HT, the impact energies decreased significantly, especially those of the SA specimens. For example, the impact energy of the P specimen dropped from 86 J to 20 J after SA treatment. The roles of HT and BD in the impact toughness will be discussed in detail in Section 3.4.

### 3.3. Fracture Surface

**Figure 9:**
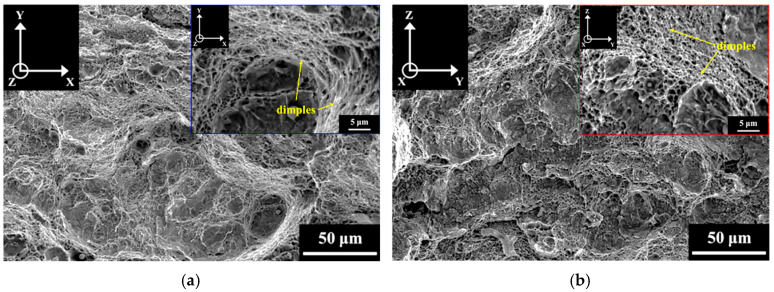
The fracture surfaces of (**a**) A-V and (**b**) A-P specimens after impact test.

**Figure 10:**
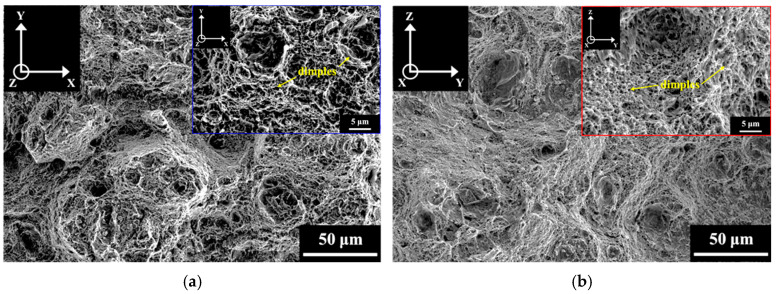
The fracture surfaces of (**a**) S-V and (**b**) S-P specimens after impact test.

**Figure 11:**
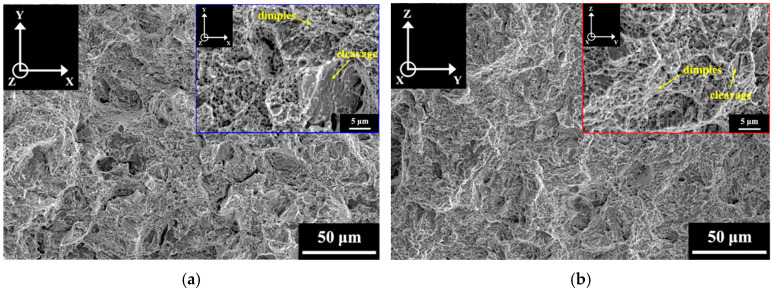
The fracture surfaces of (**a**) SA-V and (**b**) SA-P specimens after impact test.

The impact fracture surfaces of the A-V and A-P specimens after impact tests are shown in Figure 9. The results clearly show dimples in both the A-V and A-P specimens, indicating that the major impact fracture mode of the A specimens was ductile. Figure 10 and Figure 11 display the impact fracture surfaces of S and SA specimens, respectively. Only dimples were found on the fracture surfaces of the S-V and S-P specimens. However, the impact fracture surfaces of the SA-V and SA-P specimens were divergent from those of A and S specimens. As shown in Figure 11, the SA specimens exhibited mixed ductile and brittle fracture behaviors after impact tests. Other than dimples, cleavage (indicated by arrows) was also identified in the SA specimens. Furthermore, Figure 9, Figure 10 and Figure 11 demonstrate that the BD did not play an obvious role in the impact fracture behaviors of the A, S, and SA specimens.

### 3.4. The Effects of HT and BD on Impact Toughness

In the literature, inconsistent effects of S and SA treatments on the amounts of austenite in SLM Corrax steels have been reported [13,14,21,32,33,34]. The austenite content can be decreased [14,21,32,34] or increased [33] after S treatment, and SA treatment can also increase [13,14,32,34] or decrease [21,33] the fraction of austenite. In this study, Figure 3, Figure 4 and Figure 5 and Table 1 show that the amount of austenite in the A specimen was reduced after S treatment. However, after SA treatment, the austenite content was obviously increased because of the formation of reverted austenite. However, the impact energies were much decreased after the SA treatment, as shown in Figure 8. This trend demonstrated that the fraction of austenite did not play a decisive role in the impact toughness. It is known that the density (or porosity) affects the mechanical properties of SLM alloys [5,12,13]. The Archimedes densities of the A-P, A-V, S-P, S-V, SA-P, and SA-V specimens were similar [14]. The densities were not apparently changed by the HT or BD, so the influences of density on the mechanical performances can be excluded.

In general, the impact energy of a metallic material relates to its strength and elongation [21,22,28]. The tensile properties of the SLM Corrax (or CX) steels have been investigated in several studies [13,14,21,31,32]. In the A state of SLM Corrax (or CX), the UTS ranges from 1043 MPa to 1168 MPa, and the tensile elongation ranges from 13.3% to 18.4% [13,14,21,31,32]. In the S condition, the UTS and tensile elongation are, respectively, 926–1129 MPa and 12.7–14.4% [14,21,32]. Both the UTS and tensile elongation of S specimens are lower than those of A specimens, leading to their lower impact energies. In the SA state of SLM Corrax (or CX), the UTS and tensile elongation are 1589–1683 MPa and 7.3–12.4%, respectively [13,14,21,32]. The SA treatment increases the UTS of SLM Corrax (or CX) by ~48% but decreases the elongation by ~38% [13,14,21,31,32]. In this study, however, the impact energies of the SA specimens were ~75% lower than those of the A specimens, as shown in Figure 8. This trend indicates that the impact toughness of SLM Corrax steel is more closely related to its tensile elongation than to its UTS.

The SA treatment can improve the strength of SLM Corrax (or CX) by dislocation hardening and precipitation hardening [15,35]. After SA treatment, dislocation hardening and precipitation hardening, respectively, contributed 866 MPa and 370 MPa to the YS [35]. However, although the SA treatment significantly strengthened the martensitic matrix, it also embrittled it. The impact fracture surfaces in Figure 9, Figure 10 and Figure 11 show that, irrespective of HT and BD, the ductile fracture mode dominated the impact fractures of the SLM Corrax specimens. However, the SA specimens exhibited greater brittleness, as indicated by the presence of cleavage fracture. Therefore, the lower impact energies of SA specimens were due to the increased brittleness of the martensitic matrix.

SLM Corrax or CX steels are mainly applied as mold materials. The requirements of a mold material include high hardness and YS and moderate ductility and impact toughness. According to the results in the literature [21,22] and this study, the A specimens presented lower hardness and YS but the highest impact energy. The S specimens also exhibited lower hardness and YS. The SA specimens exhibited the highest hardness and YS along with moderate ductility and impact energy. Thus, in the mold industry, SLM Corrax steel molds are generally applied in the SA condition.

Figure 8 shows that the impact energies of SA-P and SA-V specimens were 20 J and 17 J, respectively, indicating that the anisotropy in impact toughness of SA specimens was merely 3 J, which is an advantage for the stability of an SLM Corrax steel mold during service. This phenomenon can be ascribed to the weak texture in SLM Corrax steels, as shown in Figure 5. Sanjari et al. [36] found that the crystallographic texture did not cause the anisotropic tensile properties of SLM CX. Moreover, Wu et al. [14] indicated that in SLM Corrax steels, weak textures were identified in the A, S, and SA conditions regardless of the BD, but these textures do not result in anisotropic tensile performances. Consequently, the impact toughnesses of SLM Corrax steels were dominated by the material condition. BD played only a minor role in the impact energies of SLM Corrax steels due to their weak textures.

## 4. Conclusions

1. According to the EBSD analyses, the matrices of the A-V, S-V, and SA-V specimens were all composed of lath martensite. After S treatment, the austenite content was slightly reduced. However, after SA treatment, the fraction of austenite was obviously increased. The EPMA results indicated that the metallic elements were uniformly distributed in the A-V specimens, and no elemental segregation was observed at the analyzed magnification.
2. The impact energies and apparent hardnesses of SLM Corrax steels were dominated by the HT. The S treatment simultaneously decreased the impact energies and apparent hardnesses. The SA treatment increased the apparent hardnesses but decreased the impact energies. BD played a minor role in both the impact energies and apparent hardnesses.
3. In the A and S specimens, the impact fracture surfaces consisted of only ductile fracture features. After the SA treatment, the impact fracture surfaces exhibited a mixture of ductile and brittle fracture modes. The lower impact energies of the SA specimens can be mainly attributed to the less ductile martensitic matrix. The amount of austenite did not greatly influence the impact energy.
4. The impact energies of SA-P and SA-V specimens were, respectively, 20 J and 17 J. The anisotropy in impact toughness of SA specimens was low due to its weak texture.

## Figures and Tables

**Table 1:** The EBSD quantitative results in various material conditions [14].

Material Condition	Building Direction	Plane Analyzed	Grain Size of Martensite Phase (µm)	Percentage of Austenite (%)
A	P	XY	1.5	2.3
		YZ	1.7	1.5
A	V	XY	2.1	0.1
		YZ	1.9	0.1
S	P	XY	1.5	0.4
		YZ	1.6	0.2
S	V	XY	1.8	0
		YZ	1.7	0.1
SA	P	XY	1.6	4.5
		YZ	1.3	3.7
SA	V	XY	1.6	7.5
		YZ	1.5	7.5

**Table 2:** EPMA quantitative analyses in an A-V specimen.

No.	Weight Percentage (wt.%)					
	Fe	Cr	Ni	Al	Mo	Total
1	75.9	12.5	8.73	1.45	1.42	100
2	75.4	12.6	9.21	1.45	1.33	100
3	75.6	12.6	8.84	1.55	1.37	100
4	76.2	12.5	8.46	1.46	1.33	100
5	75.4	12.7	8.97	1.5	1.42	100
6	75.3	12.6	9.1	1.55	1.41	100

## Data Availability

The raw data required to reproduce these results cannot be shared at this time, as the data also form part of an ongoing study.
